# Genotypic and phenotypic characteristics of 12 chinese children with glycogen storage diseases

**DOI:** 10.3389/fgene.2022.932760

**Published:** 2022-08-29

**Authors:** Rui Dong, Xuxia Wei, Kaihui Zhang, Fengling Song, Yuqiang Lv, Min Gao, Dong Wang, Jian Ma, Zhongtao Gai, Yi Liu

**Affiliations:** ^1^ Pediatric Research Institute, Children’s Hospital Affiliated to Shandong University (Jinan Children’s Hospital), Jinan, China; ^2^ Shandong Provincial Clinical Research Center for Children’s Health and Disease, Jinan, China; ^3^ Gastroenterology, Children’s Hospital Affiliated to Shandong University (Jinan Children’s Hospital), Jinan, China; ^4^ Children’s Health Department, Children’s Hospital Affiliated to Shandong University (Jinan Children’s Hospital), Jinan, China

**Keywords:** glycogen storage diseases, mutation, whole exome sequencing, GAA gene, AGL gene, PHKA2 gene, PYGL gene

## Abstract

**Background:** Glycogen storage diseases (GSDs) are known as a group of disorders characterized by genetic errors leading to accumulation of glycogen in various tissues. Since different types of GSD can sometimes be clinically indistinguishable, next generation sequencing is becoming a powerful tool for clinical diagnosis.

**Methods:** 12 patients with suspected GSDs and their parents were enrolled in this study. The clinical and laboratory data of the patients were reviewed. Causative gene variants were identified in the patients using whole exome sequencing (WES) and verified by Sanger sequencing.

**Results:** Genetic testing and analysis showed that 7 patients were diagnosed with GSD II (Pompe disease), 2 patients with GSD III, 1 patient with GSD VI, and 2 patients with GSD IXα. A total number of 18 variants were identified in 12 patients including 11 variants in *GAA* gene, 3 variants in *AGL* gene, 2 variants in *PYGL* gene and 2 variants in *PHKA2* gene, of which 9 variants were reported and 9 variants were novel. SIFT, Polyphen-2, Mutation Taster, and REVEL predicted the novel variants (except *GAA* c.1052_1075 + 47del) to be disease-causing. The 3D structures of wild/mutant type GAA protein were predicted indicating that variants p. Trp621Gly, p. Pro541Leu, p. Ser800Ile and p. Gly293Trp might affect the proteins function via destroying hydrogen bonds or conformational constraints. Neither liver size nor laboratory findings allow for a differentiation among GSD III, GSD VI and GSD IXα.

**Conclusion:** Our study expanded the variation spectrum of genes associated with GSDs. WES, in combination with clinical, biochemical, and pathological hallmarks, could provide accurate results for diagnosing and sub-typing GSD and related diseases in clinical setting.

## Introduction

Glycogen storage diseases (GSDs) as the innate defects of carbohydrate metabolism are caused by enzyme deficiency in the glycogenolysis or gluconeogenesis (Liang et al., 2020). There are 19 types of GSD, named in Roman numerals from 0 to XV (GSD I was divided into 4 subtypes numbered a-d), classified by enzyme deficiency and affected tissue. In aggregate, GSDs are not considered rare diseases, but every subtype of the disorder are (Molares-Vila et al., 2021). The overall estimated GSD incidence is 1 case per 10,000 live births (Beyzaei et al., 2020). Like most metabolic diseases, the vast majority of GSDs are inherited in an autosomal recessive (AR) pattern, except for X-linked type IXα (Brown et al., 2015; Szymanska et al., 2021). In GSD Ⅱ, the symptoms are caused by mutations in *GAA* gene and in GSD III, *AGL* gene. In GSD VI, the clinical manifestations are associated with mutations in *PYGL* gene and in GSD IXα, *PHKA1* and *PHKA2* gene (De Vito et al., 2019; Kim et al., 2020; Perveen et al., 2020; Grunert et al., 2021a).

GSDs are a group of clinically and genetically heterogenous diseases. The clinical manifestations of GSDs range from almost no symptoms to severe cardiac dysfunction, respiratory insufficiency, and sudden death (Molares-Vila et al., 2021). GSDs are divided into hepatic, muscle and mixed types according to glycogen accumulation in tissues (Lu et al., 2016; Zhao et al., 2019; Eghbali et al., 2020; Szymanska et al., 2021). The basic clinical symptoms of hepatic GSD are hypoglycemia and hepatomegaly. The characteristic feature of muscle GSD is progressive muscle pathology, including exercise-induced muscle weakness. In both types of GSD, there is also hypertransaminasemia. The different types of GSD are sometimes clinically indistinguishable, especially in hepatic GSDs (Choi et al., 2016). Early diagnosis and effective treatment of GSDs is necessary to prevent adverse outcomes. Clinical signs and symptoms and laboratory parameters such as hypoglycemia, hypertransaminasemia and increased triglycerides level are helpful in GSDs diagnosis. Liver biopsy is an invasive test to assess liver cell enzyme activity. Gene sequencing and genetic analysis, a technology that can provide precise diagnosis and procreation guidance and are more acceptable to patients, is increasingly used in the diagnosis of genetic diseases, including GSD (Shen and Shi, 2019; Fang et al., 2021; Huang et al., 2021; Li et al., 2021; Szymanska et al., 2021).

In this study, by clinical and next-generation sequencing analysis of 12 patients with suspected GSDs, we aimed to give them molecular diagnosis of GSD and determine the spectrum of genetic analysis specific to our cohort. As a result, we found 18 variants in *GAA*, *AGL*, *PHKA2* and *PYGL* gene related to GSD and the 12 patients were diagnosed with GSD types II, III, VI, and IXα respectively.

## Materials and methods

### Study cohort

A total of 12 hospitalized children (6 female, 6 male) in unrelated families with clinically suspected GSD were recruited for this study in Children’s Hospital Affiliated to Shandong University (Jinan Children’s Hospital) from November 2018 to October 2021. All patients from the Han Chinese population in Shandong Province, China were examined and diagnosed by experienced pediatric specialists from the Neurology or Gastroenterology Department. The clinical records of 12 patients with GSD were retrospectively reviewed.

### Genetic analysis

Blood samples were obtained from the patients and their parents, and DNA was extracted form peripheral blood leukocytes using QIAamp DNA Blood Midi Kit (Qiagen, Shanghai, China). Mutation screening of the patients was applied by Whole exome sequencing (WES) with the Human Exome Probes P039Exome (MyGenostics, Beijing, China) on the Illumina NovaSeq5000 platform (Illumina, United States). The obtained mean exome coverage was more than 95% (>10X coverage; mean depth of over 100X). Paired-end alignment was performed with version GRCh37/hg19 of the human genome on Burrows-Wheeler Aligner software (BWA Version: 0.7.10). Variant frequencies were determined in thousands of genomes (1000 Genomes, http://www.1000genomes.org), ExAC (http://exac.broadinstitute.org/), Exome Variant Server (EVS, http://evs.gs.washington.edu/EVS) and in-house database to remove common variants (suballelic frequency >5%). Frameshift variants, missense variants, premature stop-gain, initiation codon loss, and typical splicing site changes were prioritized. Effect on protein function of variations were predicted by SIFT, PolyPhen-2, MutationTaster, and REVEL. Descriptions of variants in Human Gene Mutation Database (HGMD, http://www.hgmd.cf.ac.uk) and ClinVar (http://www.ncbi.nlm.nih.gov/clinvar) also were referenced. Swiss-Pdb Viewer 4.1 software was utilized to generate the 3D protein plots of wild-type and mutant type for novel missense variants. The variants identified in this study were classified according to the 2015 American College of Medical Genetics and Genomics (ACMG) guidelines (Richards et al., 2015).

### Validation of gene mutations

The target variants screened from WES were verified by Sanger sequencing in parents and probands using ABI Prism 3700 automated sequencer (Applied Biosystems, Foster City, CA).

### Statistical analysis

Statistical analysis was performed on SPSS 24.0 software (SPSS Inc. Chicago IL, USA). The Median and Interquartile range (IQR) of onset age and diagnosis age was calculated. The Shapiro–Wilk test was used to test for normality of the data. For data with non-normal distribution, Kruskal-Wallis H test was used for multiple group comparisons and Mann-Whitney U test for two group. *P* < 0.05 (two-tailed) were considered statistically significant.

## Results

### Clinical manifestations and laboratory tests

Clinical details were available for 12 patients. The age, sex, physical examination and laboratory findings at the time of diagnosis were recorded and showed in Table 1. The average age of onset was 11.5 ± 36.5 months, and the age of diagnosis was 22.5 ± 38.5 months. The initial symptoms of patients in our cohort were hypertransaminases, motor retardation due to muscle weakness, hypertrophic cardiomyopathy, and abdominal distension. Patients 1-7 had elevated liver enzymes and muscle weakness and were classified to mixed GSD, while patients 8–12 had only a liver-related phenotype without muscle involvement and were classified to hepatic GSD.

**TABLE 1:** | The clinical and laboratory features of the Chinese patients with GSD.

Patient	—	P1	P2	P3	P4	P5	P6	P7	P8	P9	P10	P11	P12
Diagnosis	—	GS II	GSD II	GSD II	GSD II	GSD II	GSD II	GSD II	GSD III	GSD III	GSD VI	GSD IXα	GSD IXα
Gender	—	F	F	M	F	F	F	M	F	M	M	M	M
Age	—	2m24d	7m9d	3y2m	2y1m	1y8m	13y3m	7m7d	1y	1y	2y8m	4y4m	4y2m
Age at the onset	—	Postnatal	Postnatal	2y	Postnatal	9m	12y9m	Postnatal	1y	11m	2y8m	3y2m	4y
Initial symptoms	—	HC	ET	MW	LB	MW	MW	HC	AD	AD	ET	ET	ET
Clinical symptoms	Motor development delay/muscle weakness	+	+	+	+	+	+	+	−	−	−	−	−
	Hypertrophic cardiomyopathy	+	+	−	+	+	NA	+	−	−	−	NA	NA
	Hepatomegaly	NA	−	+	+	−	NA	−	+	+	+	+	+
	(mm below costal)	NA	NA	51	37	NA	NA	NA	72	83	53	68	70
	Short stature	−	−	+	+	−	NA	−	−	−	−	+	+
	Malnutrition	+	+	−	−	−	NA	−	−	−	−	−	−
Lab tests	Alanine aminotransferasea	119↑	161↑	148↑	150↑	305↑	NA	82↑	833↑	469↑	610↑	46↑	516↑
	Aspartate aminotransferasea	209↑	362↑	227↑	227↑	380↑	227↑	146↑	1131↑	636↑	999↑	78↑	516↑
	Lactate dehydrogenasea	586↑	964↑	667↑	599↑	817↑	589↑	1042↑	984↑	532↑	955↑	251↑	400↑
	Hydroxybutyrate dehydrogenasea	500↑	886↑	620↑	486↑	721↑	467↑	1015↑	456↑	301↑	468↑	207↑	251↑
	Glutamyl transpeptidasea	53.8↑	15.8	16	14.2	9.9	NA	15.2	100.6↑	201.2↑	66.2↑	28.7	243.3↑
	Ccreatine kinasea	624↑	538↑	717↑	815↑	820↑	2049↑	388↑	154	191	79	80	35
	Glycemiab	4.13	4.01	5.3	5.42	4.59	NA	6.5	1.09↓	0.86↓	3.3↓	3.53↓	2.64↓
	Lactic acidb	NA	2.1	NA	1.3	0.7	NA	1.1	1.5	2.8↑	3.9↑	NA	4.2↑
	Triglyceridesb	NA	2.00↑	NA	NA	0.86	NA	NA	3.36↑	8.36↑	NA	1.90↑	3.43↑
	Cholesterolb	3.20	4.20	7.59↑	7.48↑	4.63	NA	2.88	4.84	4.46	2.87	4.80	5.23

M, male; F, female; HC, Hypertrophic cardiomyopathy; ET, Hypertransaminase; MW, muscle weakness; LB, labored breathing; AD, abdomina distention. NA: not available; y, year(s); m, month(s); d, day(s); “+”: Present; “-”: Absent

### Candidate variants

Compound heterozygous/homozygous variants in *GAA* gene (P1-P7), *AGL* gene (P8, P9), or *PYGL* gene (P10) were detected in our patients, respectively. Patients 11–12 were detected as heterozygotes for the PHKA2 gene variant. Totally 18 different variants in four GSD related genes were found (variants c.2662G>T, c.1798C>T, and c.1622C>T of *GAA* gene were detected twice), of which 9 variants were reported and 9 variants were novel. The variants in the patients were inherited from their carrier parents, except for 2 male patients with *PHKA2* gene variant on the X chromosome were from their mother. GSD type, variation location, variation, amino acid variation, variant type, and mutation analysis results of the variations are given in Tables 2 , 3.

**TABLE 2:** | Mutations analyzed by WES and classified according to the ACMG guidelines.

Patient	Gene	Het/Hom	Mutation 1					Mutation 2				
			Location	DNA change	Protein change	Variant type	Pathogenic evaluation according to ACMG	Location	DNA change	Protein change	Variant type	Pathogenic evaluation according to ACMG	



P1	GAA	Chet	exon19	c.2662G>T	p.Glu888X	Nonsense	P	exon13	c.1861T>G	p.Trp621Gly	Missense	LP	
P2	GAA	Chet	exon14	c.1933G>A	p.Asp645Asn	Missense	P	exon13	c.1798C>T	p.Arg600Cys	Missense	P	
P3	GAA	Chet	exon11	c.1622C>T	p.Pro541Leu	Missense	LP	exon17	c.2399G>T	p.Ser800Ile	Missense	LP	
P4	GAA	Chet	exon6	c.1052_1075+47del	—	Splicing	P	exon11	c.1622C>T	p.Prp541Leu	Missense	LP	
P5	GAA	Chet	exon13	c.1798C>T	p.Arg600Cys	Missense	P	exon11	c.1557G>A	p.Met519Ile	Missense	LP	
P6	GAA	Chet	exon6	c.1004G>A	p.Giy335Glu	Missense	P	exon11	c.1634C>T	p.Pro545Leu	Missense	LP	
P7	GAA	Chet	exon19	c.2662G>T	p.Glu888X	Nonsense	P	exon5	c.877G>T	p.Gly293Trp	Missense	LP	
P8	AGL	Hom	intron30	c.4161+2T>G	—	Splicing	P	—	—	—	—	—	
P9	AGL	Chet	exon30	c.4119delT	p.Cys1373Trpfs*11	Frame shift	P	exon32	c.4284T>G	p.Try1428X	Nonsense	P	
P10	PYGL	Chet	intron12	c.1518+1G>A	—	Splicing	P	exon6	c.730C>T	p.Leu244Phe	Missense	LP	
P11	PHKA2	Hemi	exon32	c.3507_3520del	p.Gln1169Hisfs*29	Frame shift	P	—	—	—	—	—	
P12	PHKA2	Hemi	exon9	c.883C>G	p.Arg295Gly	Missense	LP	—	—	—	—	—	

The variants are described using NM_000152 for GAA, NM_000642 for AGL, NM_002863 for PYGL and NM_000292 for PHKA2 transcript reference sequences. Chet, compound heterozygous; Hom, homozygous; Hemi, hemizygous; P, Pathogenic; LP, Likely Pathogenic; novel variants: Bold font.

**TABLE 3:** | Chr positions of the variants in GAA, AGL, PHKA2 and PYGL gene.

Patient no.	Gene	Mutation 1		Mutation 2	
		Position	c.DNA	Position	c.DNA
1	GAA	chr17:78092467	c.2662G>T	chr17:78086483	c.1861T>G
2	GAA	chr17:78086719	c.1933G>A	chr17:78086420	c.1798C>T
3	GAA	chr17:78084810	c.1622C>T	chr17:78091466	c.2399G>T
4	GAA	chr17:78082185-78082256	c.1052_1075+47del	chr17:78084810	c.1622C>T
5	GAA	chr17:78086420	c.1798C>T	chr17:78084745	c.1557G>A
6	GAA	chr17:78082137	c.1004G>A	chr17:78084822	c.1634C>T
7	GAA	chr17:78092467	c.2662G>T	chr17:78081617	c.877G>T
8	AGL	chr1:100379296	c.4161+2T>G	—	—
9	AGL	chr1:100379251-100379252	c.4119delT	chr1:100381990	c.4284T>G
10	PYGL	chr14:51381418	c.1518+1G>A	chr14:51387716	c.730C>T
11	PHKA2	chrX:18912338- 18912352	c.3507_3520del	—	—
12	PHKA2	chrX:18958148	c.883C>G	—	—

The nine novel variants were determined after checked in all available databases and publications, including 1000 Genomes, ExAC, gnomAD, in-house databases and published articles. The scores of SIFT, Polyphen-2, Mutation Taster, and REVEL predicted that the novel variants (except c.1052_1075 + 47del) have a deleterious effect on the function of the protein (Table 4). No predictions of impact were available for variant c.1052_1075 + 47del by bioinformatics algorithms. In the novel variants, 3 splicing and 1 frameshift mutations was classified as pathogenic, 5 missense mutations as likely pathogenic according to the ACMG guidelines (Richards et al., 2015).

**TABLE 4:** | Effect on protein function of the novel variations predicted by SIFT, PolyPhen-2, MutationTaster, and REVEL.

Patient	Gene	Variant	Variant type	1000 Genome	ExAc	gnomAD	SIFT		Polyphen-2		Mutation taster		REVEL	
							Score	P	Score	P	Score	P	Score	P
P1	GAA	p.Trp621Gly	Missense	—	—	—	0	D	1	PD	1	DC	0.777	D
P3	GAA	p.Pro541Leu	Missense	—	—	—	0.005	D	0.994	PD	1	DC	0.892	D
P3	GAA	p.Ser800Ile	Missense	—	—	—	0.009	D	0.989	PD	1	DC	0.769	D
P7	GAA	p.Gly293Trp	Missense	—	—	—	0	D	1	PD	1	DC	0.899	D
P12	PHKA2	p.Arg295Gly	Missense	—	—	—	0	D	0.998	PD	1	DC	0.992	D
P8	AGL	c.4161+2T>G	Splicing	—	—	—	NA	NA	NA	NA	1	DC	NA	NA
P10	PYGL	c.1518+1G>A	Splicing	—	—	—	NA	NA	NA	NA	1	DC	NA	NA
P11	PHKA2	c.3507_3520del	Frame shift	—	—	—	NA	NA	NA	NA	1	DC	NA	NA

P, Prediction; D, Damaging; PD, Probably_damaging; DC, Disease_causing; NA: not available.

**FIGURE 1:**
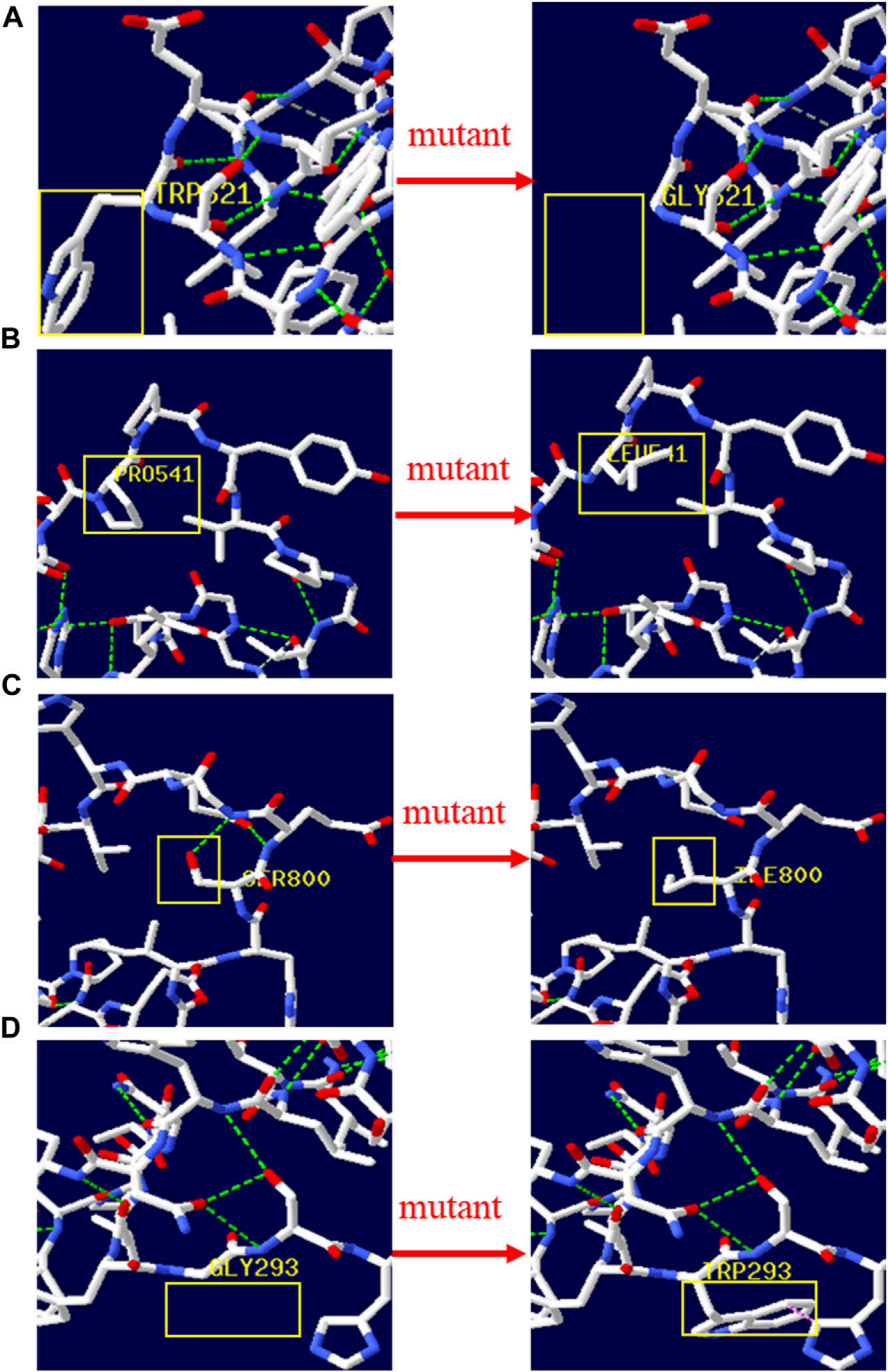
3D structure of wild type and mutant type of novel variants in GAA. The yellow boxes marked the sites of wild‐type Gly293, Prp541, Trp621, Ser800 and mutated Gly621, Leu541, Ile800, Trp293. The 3D protein structures of novel variants in GAA gene were predicted by Swiss‐Pdb Viewer 4.1 indicating the missense mutations might affect the proteins function via destroying hydrogen bonds, or conformational constraints which resulted in the proteins into disordered extended structures.

**FIGURE 2:**
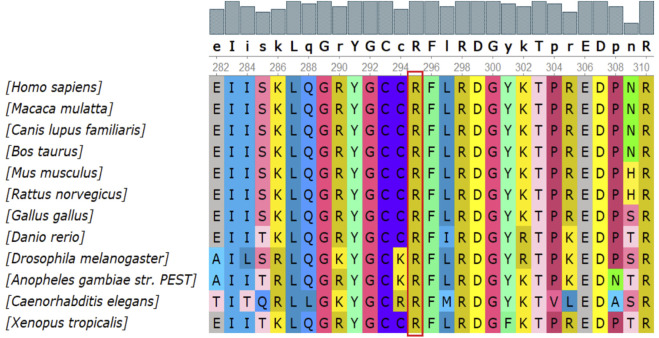
Conservation analysis of the p.Arg295Gly mutation in *PHKA2*. Silico analysis of p.Arg295 in *PHKA2* shows the site highly conservative in different species of human, Macaque, canine, Cattle, etc.

There were 4 mutations of *GAA* and 1 of *PHKA2* in the novel missense variants. The 3D protein structures of *GAA* with novel missense variants were predicted by Swiss-Pdb Viewer 4.1 indicating the variants might affect the proteins function via destroying hydrogen bonds, or conformational constraints which resulted in the proteins into disordered extended structures (Figure 1). The protein structure of *PHKA2* with p. Arg295Gly was not constructed because there was no suitable model in SWISS-MODEL database. Silico analysis showed that the p. Arg295 in *PHKA2* was highly conservative in different species of human, Macaque, canine, Cattle, et al. (Figure 2).

### Genetic diagnosis and clinical analysis

Gene sequencing showed that 7 patients were diagnosed with GSD II, 2 patients with GSD III, 1 patient with GSD VI, and 2 patients with GSD IXα. In the study group, the most frequent subtype of GSD was type Ⅱ with 58.3% (n = 7), followed by GSD III (n = 2) and IXα with 16.7% (n = 2).

Seven GSD II patients were divided into infantile-onset Pompe disease (IOPD) (P1, P2, P7) group and late-onset Pompe disease (LOPD) (P3-P6) group according to appearance cardiomyopathy within/out 12 months and age of onset (Molares-Vila et al., 2021). The initial symptoms of GSD II patients were Hypertrophic cardiomyopathy (66.6%) and Hypertransaminase (33.3%) in IOPD group, while exercise-induced muscle weakness (75.0%) and labored breathing (25.0%) in LOPD group. In our study, laboratory findings of IOPD and LOPD were not significantly different except for CK values (*p* > 0.05).

There were 5 patients with hepatic GSD in our study, including 2 patients with GSD III, 1 patient with GSD VI, and 2 patients with GSD IXα. Hepatomegaly, hypoglycemia and hypertransaminase were observed in all patients, hypertriglyceridemia in 80% (4/5) patients and hyperlactate in 60% (3/5, 1 undetected) patients. The first and main symptom of patients (P8, P9) with GSD III was progressive abdominal distention (100%) due to enlarged liver. The main symptom observed in the patient (P10-P12) with GSD VI or IXα was hypertransaminasemia (100%) detected on laboratory examination accompanied by hepatomegaly. Neither liver size nor laboratory findings allow for a differentiation among GSD III, GSD VI and GSD IXα (*p* > 0.05). At diagnosis, both patients with GSD IX had a height shorter than -2 SDS, while growth retardation was not found in GSD III and GSD VI patients. Between GSD II group and the hepatic GSD group, significant differences in liver size and partial laboratory findings (glutamyl transpeptidasea, hydroxybutyrate dehydrogenasea, creatine kinasea, Glycemia, Lactic acid) were found.

### Treatment and follow-up

Of seven GSD II patients, six received enzyme replacement therapy (ERT) (Myozyme^®^) injection, 4 of whom responded to treatment, 2 were discontinued due to death with severe myocardial hypertrophy and pulmonary hypertension and hypersensitivity, and 1 patient lost visit. Two patients with GSD III received oral raw cornstarch and increased dietary protein after diagnosis. During treatment, they occasionally experienced convulsions due to fasting hypoglycemia, and developed symptoms of short stature and delayed motor development. In patients with GSD VI and IXα, after receiving oral raw cornstarch, the clinical symptoms basically disappeared except for the hypertransaminases.

## Discussion

### Diagnosis and distribution of GSDs

The molecular mechanism of GSD is variation in genes encoding key enzymes in glycogenolysis or gluconeogenesis (Ying et al., 2020). With the development of gene sequencing technology, identification of variants by next-generation sequencing (NGS) has partially replaced traditional invasive examinations, such as liver biopsy, muscle biopsy, etc. As the accurate standard for diagnosis of GSD (Sperb-Ludwig et al., 2019; Beyzaei et al., 2020; Szymanska et al., 2021). In the present study, 18 different heterozygous candidate mutations (9 novel and 9 reported) were identified in 12 Chinese GSD families: 7 families carrying *GAA* mutations (7/12, 58.3%), 2 carrying *AGL* mutations (2/12, 16.7%), 1 carrying *PYGL* mutation (1/12, 8.3%), and 2 carrying *PHKA2* mutations (2/12, 16.7%). According to the ACMG guidelines (Richards et al., 2015), 4 of the 9 novel variants was classified as pathogenic, 5 classified as likely pathogenic, and all of them have not been reported in any human gene mutation databases or publications (Table 2). Early and accurate diagnosis of GSD is an important step in realizing personalized management, helping physicians to formulate the best treatment plan for patients and minimizing adverse outcomes (Retterer et al., 2016; Beyzaei et al., 2021; Unnisa et al., 2022). The median age of the 12 patients diagnosed in this study was 22 months (including 5 cases less than or equal to 1 year old), meaning that an earlier diagnosis and treatment is more conducive to reducing the risk of organ damage progression and death.

It has been known the geographical distribution of GSD subtypes and genotype was different. In a cohort of Iranian, GSD III (*AGL*), GSD IX (*PHKB* and *PHKG2*) was the major type of GSD (Beyzaei et al., 2021). In Turkish, the genetic analysis showed that the highest proportion of GSDs was type III with 39.5% (n = 15), followed by type I with 36.8% (n = 14) (Cakar et al., 2020). While in Spain, Vega et al. reported that more than three-quarters of patients with GSDs were type III or IXα (Vega et al., 2016). In our study cohort, more than half of the patients were diagnosed as GSD II which suggested that type II may be the most common subtype in the Chinese GSD population.

### Patients with GSD II

GSD II (MIM# 232300), also known as Pompe disease, caused by deficiency of the enzyme acid α-glucosidase encoded by *GAA*, presents as a progressive myopathy due to accumulation of glycogen in lysosomes and cell destruction (van Capelle et al., 2016). GSD II presents as a broad clinical spectrum of phenotypes with considerable variation in age of onset, symptom/presenting sign, degree of severity, organ involvement, and nature history (Holzwarth et al., 2022). In our study, 7 patients were detected compound heterozygous mutations in *GAA* gene with an age ranging from birth to 12 years old. Three patients (P1, P2, P7) manifesting generalized myasthenia and hypertrophic cardiomyopathy within the first months of life were IOPD. Two of them also showed severe malnutrition. Four patients (P3-P6) who presented as progressive limb-girdle muscle weakness with hyperCKemia were LOPD. Noteworthily, although P4 developed dyspnea shortly after birth due to respiratory muscle involvement, but she had relatively mild myocardial damage and was classified as LOPD (Hout et al., 2003; Kishnani et al., 2006; Chawla et al., 2022).

Of the *GAA* variants reported worldwide, c.-32-13T>G is the most common, especially in Caucasian populations. In Asia Pacific, p. Asp645Glu, p. Trp746Cys, p. Gly576Ser, p. Glu888X were most reported (Bergsma et al., 2019; Reuser et al., 2019; Zhao et al., 2019). While in our study, p. Glu888X were detected in P1 and P7, and no other Asia-Pacific high-frequency variants were found. Previous genotype-phenotype correlation research have shown that variants p. Glu888X, p. Asp645Asn, and p. Arg600Cys were associated with IOPD (Reuser et al., 2019). The conclusion was confirmed in our study that 4 patients (P1, P2, P5, and P7) carried one or both of the above variants manifest as early-onset or severe symptom.

We identified 6 reported variants and 5 novel variants of *GAA* in seven patients with GSD II. Four of the new variants were missense variants and all of them located in the GAA protein domain: p. Gly293Trp maps to the N-terminal Beta Sheet domain, p. Prp541Leu and p. Trp621Gly maps to the catalytic GH31 domain, p. Ser800Ile Maps to the Proximal Beta Sheet field. The four variants were predicted to be disease-causing by SIFT, Mutation taster, REVEL and probably-damaging by Polyphen. The 3D protein structures of GAA protein were predicted and indicated that the novel missense mutations might affect the proteins function *via* destroying hydrogen bonds, or conformational constraints which resulted in the proteins into disordered extended structures (Figure 1). In addition, p. Gly293Arg, resulting in co-located but different amino acid changes with p. Gly293Trp, has been described in two LOPD patients and identified as pathogenic (Loscher et al., 2018). Another new variant was c.1052_1075 + 47del, a splice variant lacking 71 nucleotides spanning exon 6 and intron 6 that could theoretically affect normal mRNA splicing. However, bioinformatics algorithms were unable to predict the effect of this variant. According to ACMG criteria, four novel missense variants were classified as probable pathogenic variants and one novel splice variant was classified as pathogenic.

### Patients with GSD III, VI and IXα

GSD III (*AGL*, MIM# 232400), VI (*PYGL*, MIM# 232700) and IXα (*PHKA2*, MIM# 306000) belong to hepatic GSD with basic features of hepatomegaly and hypoglycemia (Beyzaei et al., 2020). They share commonalities also in other clinical signs: metabolic abnormalities, short stature, and motor development delay/muscle weakness with aging, that lead to challenging to identify the subtypes of patients with hepatic GSD, especially between VI and IX (Qu et al., 2020; Szymanska et al., 2021). However, some subtypes have been found to have specific phenotypic features, for example growth delay is more common in GSD IX patients than in VI patients and hypoglycemia was more pronounced in GSD III than GSD VI and IX (Kishnani et al., 2019; Fernandes et al., 2020; Grunert et al., 2021b). In our study, there were 2 patients of GSD III, 1 patient of GSD VI and 2 patients of GSD IXα. Hepatomegaly, hypoglycemia and hypertransaminase were observed in all patients, hypertriglyceridemia in 80% patients and hyperlactate in 60% patients. Neither liver size nor laboratory findings allow for a differentiation among GSD III, GSD VI and GSD IXα in our study, but only patients with GSD IXα had a height below -2 SDS, which was consistent with the findings of Grunert et al. (Grunert et al., 2021b). None of our patients had significant motor retardation when diagnosed. Interestingly, the initial symptom of two GSD III patients was abdominal distention due to hepatomegaly, whereas GSD VI and IX patients presented with elevated transaminases inadvertently during upper respiratory tract infection.

We identified 3 variants of *AGL* gene in patients with GSD III: p. Cys1373Trpfs*11 and p. Try1428X were detected compound heterozygous in patient 9 and already reported in Chinese population (Lu et al., 2016); c.4161 + 2T>G as a novel splicing variation was detected to be homozygous in P8 while her parents were from two unrelated families. c.4161 + 2T>G was predicted to affect normal splicing of mRNA and evaluated as disease-causing by Mutation taster. According to the standard of ACMG, c.4161 + 2T>G was classified as pathogenic mutation. Notably, the 3 variants were concentrated in the 30th intron or exon, and the 2 patients with them have similar phenotypes and onset times.

GSD VI is rare relative to other types of GSD, with an incidence of 1/60,000 to 1/85,000 (Zhan et al., 2021). As the only causative gene of GSD VI, *PYGL* gene has been recorded 80 types of mutations in the HGMD database (22 April 2022), among which missense/nonsense mutations are the most common. So far, 17 Chinese patients with GSD VI have been reported, and no *PYGL* hotspot variant has been found in this group (Lu et al., 2020; Luo et al., 2020; Zhan et al., 2021). For P10, we identified compound heterozygous point mutations in *PYGL* through WES, including a recurrent missense mutation p. Leu244Phe on exon 6 and a novel splicing mutation c.1518 + 1G>A on intron 12 (Lu et al., 2020). c.1518 + 1G>A was predicted to resulting in forming incorrect mRNA molecule and evluated as disease-causing by Mutation taster. Variant frequency was 0 in the database of 1000 Genomes, gnomAD and ExAC. According to the ACMG standard, c.1518 + 1G>A was classified as a pathogenic variant.

In liver form of GSD IXα, the disease is inherited in an X-linked recessive manner associated with *PHKA2* mutations (Fang et al., 2021; Mori et al., 2022). As of 22 April 2022, 166 *PHKA2* variants were recorded in the HGMD database, with missense/nonsense mutations (greater than 50%) being the most common, followed by small deletion variants. In this report, we detected two novel variants in *PHKA2*, c.3507_3520del (p.Gln1169Hisfs*29) and p. Arg295Gly. p. Gln1169Hisfs*29 lead to fourteen-nucleotide deletion within exon 32 of PHKA2, that seemed to result in a frameshift introducing a premature stop codon after 2602 bp of amino acid residue 1196 of 1235. The variant was predicted disease-causing by Mutation taster and evaluated as a pathogenic variant refer to ACMG guidelines. Notably, the *PHKA2* gene had a total of 33 exons, and one-fifth (34/166) of the reported variants were located in or spanned exons 30–33, suggesting that the C-terminus of the *PHKA2* was a variant-dense region. p. Arg295Gly is an unreported missense variant, but p. Arg295His (Fernandes et al., 2020) and p. Arg295Cys (Ban et al., 2003) had been described in GSD IXα patients and identified as pathogenic. Silico analysis of p. Arg295 in *PHKA2* shows the site highly conservative in different species of human, Macaque, canine, Cattle, et al. SIFT, Mutation taster and Polyphen predicted that p. Arg295Gly was damaging to protein function. Therefore, p. Arg295Gly was assessed as likely pathogenic according to ACMG criteria.

## Conclusion

This study reported 12 Chinese patients with Glycogen storage disease and identified 18 gene variants of *GAA*, *AGL*, *PHKA2* and *PYGL* (9 novel and 9 reported) by whole-exome sequencing. Our study expanded the variation spectrum of gene associated with GSDs. Next-generation sequencing was considered the preferred diagnostic method for GSD because invasive liver biopsy can be avoided.

## Data Availability

The data presented in the study are deposited in the GenBank repository, accession number OP117444-OP117449.
